# Reliability and Validity of the Chinese Version of the Nurse’s Spiritual Sensitivity Scale

**DOI:** 10.1155/jonm/9431630

**Published:** 2026-07-15

**Authors:** Zifeng Li, Xiaojing Zhou, Luhuan Yang, Fu Ni, Huiqin Liu, Yongting Wei, Yuanzhi Fu, Zuyang Xi

**Affiliations:** ^1^ Department of Traditional Chinese Medicine, The First College of Clinical Medical Science, Three Gorges University/Yichang Central People’s Hospital, Yichang, Hubei, 443003, China; ^2^ Department of Pediatrics, The First College of Clinical Medical Science, Three Gorges University/Yichang Central People’s Hospital, Yichang, Hubei, 443003, China; ^3^ Faculty of Medicine and Health Sciences, Universiti Putra Malaysia, Serdang, Selangor, 43400, Malaysia, upm.edu.my; ^4^ School of Health and Medicine, China Three Gorges University, Yichang, Hubei, 443003, China, ctgu.edu.cn; ^5^ Department of Nursing, The First College of Clinical Medical Science, Three Gorges University/Yichang Central People’s Hospital, Yichang, Hubei, 443003, China; ^6^ Hubei Key Laboratory of Ischemic Cardiovascular Disease, Yichang, Hubei, 443003, China

**Keywords:** cross-cultural adaptation, psychometrics, reliability, spiritual sensitivity, validity

## Abstract

**Background:**

Nurses’ spiritual sensitivity refers to the ability to identify the spiritual needs of patients and their families and to address these needs through ethical decision‐making in clinical practice. However, there are currently no validated instruments to measure the spiritual sensitivity of Chinese nurses.

**Aims:**

This study aimed to adapt the Nurses’ Spiritual Sensitivity Scale (NSSS) into Chinese and to evaluate its reliability and validity among Chinese nurses.

**Methods:**

After forward translation, back‐translation, cross‐cultural adaptation, and expert consultation, a Chinese version of the NSSS was developed. In a cross‐sectional study, a total of 508 nurses were recruited from two hospitals to evaluate the instrument’s psychometric properties. Content validity was assessed using the content validity index (CVI), and structural validity was evaluated using exploratory factor analysis (EFA) (*n* = 303) and confirmatory factor analysis (CFA) (*n* = 205). Reliability was examined using Cronbach’s *α* coefficient for internal consistency and the intraclass correlation coefficient (ICC) for test–retest reliability.

**Results:**

The Chinese version of the NSSS comprises 20 items across two dimensions: professional spiritual sensitivity and inner spiritual sensitivity. The item‐level CVI (I‐CVI) ranged from 0.833 to 1.000; the scale‐level CVI (S‐CVI) was 0.900, and the average S‐CVI was 0.983. EFA (*n* = 303) extracted two factors, with a cumulative variance explained of 61.658%. CFA (*n* = 205) supported a two‐dimensional structure. The overall Cronbach’s *α* coefficient of the scale was 0.927, and the test–retest reliability coefficient was 0.911. For the two dimensions, the Cronbach’s *α* coefficients were 0.943 and 0.914, and the test–retest reliability coefficients were 0.867 and 0.935.

**Conclusions:**

The Chinese version of the NSSS is a reliable and valid instrument for assessing the spiritual sensitivity of Chinese nurses.

**Implications for Nursing Management:**

The validated Chinese NSSS offers potential utility for screening training needs, evaluating educational interventions, and guiding clinical supervision in spiritual care.

## 1. Introduction

Spiritual sensitivity refers to an individual’s awareness of, and responsibility for, others’ spiritual needs and values [1, 2]. It is one of the necessary conditions for delivering spiritual care [1, 2]. In nursing, nurses’ spiritual sensitivity denotes the ability to identify the spiritual needs (e.g., beliefs, values, and emotional support) of patients and their families in clinical practice and to address these needs through ethical decision‐making [3]. As a higher level perception and reflection within spiritual nursing, nurses’ spiritual sensitivity comprises three aspects: spiritual–professional characteristics, perception of patients’ and families’ spiritual needs, and reflective engagement with patients’ and families’ religious beliefs [3]. In this study, we conceptualize nurses’ spiritual sensitivity as a measurable professional competence that integrates an attitudinal disposition with relational and clinical judgment skills. It not only encompasses the acute perception of patients’ spiritual and emotional states but also emphasizes nurses’ respectful and responsive care for patients’ spiritual needs within holistic nursing [4].

Within the biopsychosocial model of care, holistic nursing prioritizes the comprehensive fulfillment of patients’ physiological, psychological, social, and spiritual needs, and nurses’ spiritual sensitivity is a key competency for achieving this goal [3, 5]. It is important to distinguish between three interrelated concepts: (1) spirituality as a broad, culturally shaped search for meaning and purpose; (2) spiritual care as the clinical practice of addressing patients’ spiritual concerns; and (3) spiritual sensitivity as the nurse’s underlying capacity to perceive and appropriately respond to those concerns. The nurses’ spiritual sensitivity is designed to measure this latter capacity. Recent international evidence suggests that nurses’ spiritual sensitivity varies across cultural, clinical, and educational contexts. In Iran, Tavakol et al. [6] reported a moderate level of spiritual sensitivity among nurses and identified a positive association between spiritual sensitivity and compassionate care. In Türkiye, Cihan Erdoğan and Doğan observed relatively high levels of spiritual sensitivity among nurses, highlighting its foundational role in holistic and palliative care [7]. By contrast, Ghonchehpour et al. [8] found lower levels of spiritual sensitivity among nursing and midwifery students, suggesting that this competency may not be adequately developed during professional education and may require targeted educational support. Collectively, these findings indicate that spiritual sensitivity is a culturally embedded and context‐dependent competency.

The theoretical significance of spiritual sensitivity lies in its close alignment with holistic nursing, person‐centered care, culturally competent practice, and ethical nursing decision‐making. Clinically, nurses with higher spiritual sensitivity can more easily recognize patients’ spiritual distress and respect religious and cultural preferences. This sensitivity also helps them avoid potential taboos and provide emotionally supportive care [9, 10]. Such care is linked with reduced anxiety, helplessness, and existential distress. It also promotes greater patient dignity, a stronger sense of security, and increased trust in the nurse–patient relationship [4]. Spiritual sensitivity may foster improved communication and more responsive care planning by helping nurses understand patients’ values, beliefs, and sources of meaning during illness. From a management perspective, assessing spiritual sensitivity can help nurse managers identify staff training needs and design targeted educational programs. It can also enhance compassionate, patient‐centered care and promote a more humane healthcare environment [11–13].

In developing measurement tools for spiritual sensitivity, researchers have created scales tailored to different social groups (Table 1). The spiritual sensitivity for children (SSSC) by Stoyles et al. [14] focuses on children’s spiritual perception in everyday contexts. The Spiritual Sensitivity Scale (SSS) by Nokelainen et al. [15] is suitable for adolescents and adults and adopts a developmental perspective to assess spiritual sensitivity across life stages. The Spiritual Sensitivity Inventory (SSI) by Straś‐Romanowska et al. [16] targets IT practitioners and examines spiritual experience in professional settings. Although these instruments effectively assess spiritual sensitivity across various groups, they have several limitations when applied in nursing. First, they overlook the features of high‐intensity interpersonal communication inherent in nursing and fail to encompass nurses’ spiritual sensitivity in complex social situations, such as nurse–patient communication and teamwork. Second, they fail to capture the unique stressors of healthcare settings, including life‐and‐death scenarios, moral dilemmas, and emergencies. Finally, they do not reflect the nursing profession’s focus on human life and health, nor do they incorporate nursing’s hierarchical management structures and continuing education systems.

**TABLE 1:** Summary of selected instruments assessing spiritual sensitivity.

Study	Target population	Constructs assessed	Reported psychometric properties
Stoyles et al. [14]	Children	Outward focus (Items 7, 12, 15, 20, 21); inward reflective focus (Items 1, 3, 6, 8, 9, 17, 18).	The Cronbach’s *α* for the overall scale was 0.79, and for the two subscales was 0.75 and 0.57, respectively. Hierarchical cluster analysis yielded two interpretable clusters.

Nokelainen et al. [15]	Preadolescents, adolescents, and adults	Awareness sensing (Items 1, 5, 13, 17, 9); mystery sensing (Items 2, 6, 10, 14, 18); value sensing (Items 3, 7, 11, 25, 19); and community sensing (Items 4, 8, 12, 16, 20).	Preadolescents: The Cronbach’s *α* for the four subscales was 0.55, 0.61, 0.76, and 0.63, respectively. EFA indicated that the four dimensions explained 45.5% of the total variance. Adolescents: The Cronbach’s *α* for the four subscales was 0.4, 0.48, 0.45, and 0.68, respectively. EFA indicated that the four dimensions explained 37.5% of the total variance. Adults: The Cronbach’s *α* for the four subscales was 0.6, 0.58, 0.73, and 0.71, respectively. EFA indicated that the four dimensions explained 45.8% of the total variance. Combined: CFA supported a four‐dimensional structure (*x* ^2^ */df* = 4.49, *p* < 0.001, RMSEA = 0.085, SRMR = 0.049, CFI = 0.926, TLI = 0.892).

Stras‐Romanowska et al. [16]	IT users’ workplace	Wisdom, consciousness, and meaning (Items 3, 12, 20, 23, 29, 34, 38, 43, 49, 55); religiosity and faith (Items 5, 10, 14, 19, 22, 28, 37, 45, 51, 57); holism and harmony (Items 6, 11, 21, 26, 33, 48); spiritual commitment (Items 7, 15, 41, 46); esthetic sensitivity (Items 2, 9, 18, 24, 31, 53); openness to other people (Items 4, 13, 17, 27, 32, 36); and moral and ethical conscience (Items 1, 8, 16, 25, 30, 35, 39, 47, 52, 58).	The Cronbach’s *α* for the overall scale was 0.95, and for the seven subscales was 0.6, 0.7, 0.6, 0.73, 0.7, 0.72, and 0.7, respectively. CFA supported a seven‐dimensional structure (*x* ^2^ */df* = 1.7, *p* = 0.05, RMSEA < 0.08, GFI = 0.95, AGFI = 0.9).

Akbari et al. [17]	Nurses	Professional Spiritual Sensitivity (items 1–12); Inner Spiritual Sensitivity (items 13–20).	The Cronbach’s *α* for the overall scale was 0.92, and for the two subscales was 0.92 and 0.88, respectively. EFA indicated that the two dimensions explained 57.62% of the total variance. The I‐CVI ranged from 0.85 to 1.000, and the average S‐CVI was 0.903.

*Note:* AGIF, adjusted goodness of fit.

Abbreviations: CFA, confirmatory factor analysis; CFI, comparative fit index; EFA, exploratory factor analysis; GIF, goodness‐of‐fit index; RMSEA, root mean square error of approximation; SRMR, standardized root mean square residual; TLI, Tucker–Lewis coefficient.

The most widely used instrument internationally for evaluating nurses’ spiritual sensitivity is the Nurses’ Spiritual Sensitivity Scale (NSSS) developed by Akbari et al. [17]. As a country with a population of 1.4 billion, more than 5 million registered nurses, and 56 ethnic groups, China’s multicultural context requires nursing practice to address the diverse beliefs and spiritual needs of different populations [18]. However, a substantial proportion of people in China does not report any formal religious affiliation. Therefore, patients’ spiritual needs in China may not be expressed through organized religious practices or explicit faith‐based activities; instead, they may involve concerns about meaning, dignity, hope, family responsibility, suffering, death, interpersonal harmony, and existential distress. The NSSS remains suitable for cross‐cultural adaptation because its two core dimensions, nurses’ professional spiritual sensitivity, including understanding patients’ needs and providing spiritual care, and nurses’ inner spiritual sensitivity, including self‐awareness and spiritual growth, align closely with the competencies required for holistic, person‐centered nursing in China’s diverse healthcare settings. Nevertheless, because several NSSS items explicitly address faith‐related activities, their interpretation and psychometric properties warrant careful evaluation in the Chinese clinical context.

Against this backdrop, it is of substantial theoretical and practical value to translate and culturally adapt the NSSS from the Iranian context into Chinese. The resulting tool is intended to identify individual or unit‐level training needs, support program evaluation in nursing education, and enable research on spiritual care competencies across different settings. However, because the original NSSS was developed in the Iranian context, its conceptualization of spirituality is embedded in a sociocultural environment in which Islam plays an important role in shaping understandings of spirituality, illness, care, and moral responsibility. In contrast, the Chinese context is characterized by more pluralistic and often less institutionally organized forms of spirituality, shaped by Confucian, Buddhist, Taoist, folk, and secular values. Rather than being expressed primarily through formal religious language, patients’ spiritual concerns in China may manifest in issues related to family responsibility, harmony, dignity, suffering, acceptance, hope, and the search for meaning during illness. In addition, China’s clinical healthcare context, including high patient volumes, time‐limited nurse–patient interactions, limited formal spiritual care training, and the lack of routinely integrated chaplaincy services in many hospitals, may affect how nurses perceive, interpret, and respond to patients’ spiritual needs. Therefore, cultural adaptation of the NSSS required consideration not only of linguistic equivalence but also of whether the items appropriately reflected the conceptual and practical manifestations of nurses’ spiritual sensitivity in Chinese clinical settings.

This study aimed to translate, culturally adapt, and systematically evaluate the psychometric properties of the Chinese version of the NSSS. The validation strategy follows established scale adaptation guidelines, focusing on content validity, construct validity (examined through both exploratory and confirmatory factor analyses [CFA]), reliability (internal consistency and test–retest stability), and floor/ceiling effects [19]. Based on the original, empirically derived scale framework, this process has developed a suitable spiritual sensitivity assessment tool for nurses in the Chinese cultural context. The study provides a scientific basis for nursing education, clinical evaluation, and policy‐making in China, promotes cross‐cultural comparisons in spiritual nursing, and thereby comprehensively improves the quality of nursing services.

## 2. Methods and Materials

### 2.1. Study Designs and Participants

A cross‐sectional study was conducted from April 21 to 27, 2025, at two branches of a tertiary general hospital in an urban area of Hubei Province, China. A total of 508 nurses were recruited. The study was conducted in accordance with the Strengthening the Reporting of Observational Studies in Epidemiology (STROBE) Statement [20], and the psychometric evaluation of the NSSS, specifically its reliability, validity, and responsiveness, followed the Consensus‐Based Standards for the Selection of Health Measurement Instruments (COSMIN) guidelines [19]. The inclusion criteria were as follows: (1) being a licensed nurse; (2) having ≥ 1 year of work experience; and (3) providing informed consent. The exclusion criteria were nursing interns or assistant nurses. These groups were excluded because the target population consisted of registered clinical nurses who provide independent patient care and are expected to assess and respond to patients’ spiritual needs in routine clinical practice. Nursing interns are still in training and usually provide care under supervision, whereas assistant nurses may differ from registered nurses in educational background, scope of practice, professional responsibilities, and involvement in holistic nursing care. Their exclusion was intended to ensure a relatively homogeneous sample for the psychometric evaluation of the Chinese version of the NSSS.

Because the scale was translated in this study, exploratory factor analysis (EFA) and CFA were undertaken [21]. For EFA, a minimum sample size of 10 participants per item was recommended [22]. In this study, the NSSS contained 20 items. Assuming a 20% nonresponse/invalid rate, the required EFA sample size was *N* = (20 × 10)/(1–0.8) = 250. For CFA, a minimum sample size of ≥ 200 was recommended [23]. For test–retest reliability, a sample of approximately 10% of the CFA sample size is commonly recommended [24]. Given the minimum CFA sample of 200, 20 participants were thus required for the test–retest analysis. Finally, 303 participants from one branch were allocated to EFA, and 205 participants from the other branch were allocated to CFA. respectively. In addition, 20 nurses completed the questionnaire again after 2 weeks; these data were excluded from the EFA and CFA. The final recruited sample met the predetermined sample size requirements for both EFA and CFA.

### 2.2. Translation and Cross‐Cultural Adaptation Process

#### 2.2.1. Translation Process

Before translation, formal permission to adapt the NSSS was obtained via email from its original author, Professor Omolbanin Akbari. The original scale, although developed for nurses in Iran, was made available to our team in English. In accordance with internationally endorsed guidelines for the cross‐cultural adaptation of psychometric instruments, the original English version of the NSSS was translated into Chinese [25]. First, two bilingual translators produced independent drafts and then discussed them with the study team to ensure accurate phrasing of scale terms; revisions were made until consensus was reached. In line with recommendations [26], the initial translators did not perform back‐translation. Instead, two additional bilingual translators, unfamiliar with the NSSS, independently back‐translated the Chinese version. Subsequently, the back‐translated version was compared with the original English version to identify potential conceptual inconsistencies. Thereafter, the four translators and all researchers convened to review the Chinese version. Revisions were made to ensure clarity and accuracy until the translated scale was finalized.

#### 2.2.2. Cross‐Cultural Adaptation

Cross‐cultural adaptation was conducted using the Delphi method [27]. First, the translated scale was sent to a panel of six experts from relevant disciplines. The panel reviewed and refined items across four dimensions: (1) accuracy of expression; (2) appropriateness of wording/presentation; (3) appropriateness of cultural adaptation; and (4) interitem relevance. A modified 4‐point Likert scale was used for quantitative ratings, alongside open‐ended comments for qualitative feedback. Discrepancies in ratings or comments were resolved through panel discussion, with revisions decided by majority vote. After two Delphi rounds and subsequent revisions, a consensus final version of the scale was produced. Apart from the systematic substitution of “religious” with the more inclusive term “faith” to better reflect the Chinese context, no other major conceptual modifications were made.

#### 2.2.3. Pretesting

On April 7, 2025, 20 nurses participated in a questionnaire pretest. After completing the questionnaire, participants rated the clarity and intelligibility of each item and provided suggestions for improvement. Based on pretest feedback and expert opinions, the questionnaire content was optimized, and the final Chinese version of the scale for the formal survey was finalized. The pretest indicated that the items were clear and easy to understand, the response options were reasonable, the structure was concise and specific, and the overall completion burden was low, demonstrating the good applicability and feasibility of the Chinese version of the NSSS in the nursing context in China.

### 2.3. Study Tools

#### 2.3.1. Personal Information Questionnaire

General information included sex, age, education level, and years of work experience.

#### 2.3.2. NSSS

The NSSS was developed by Omolbanin Akbari et al. in 2023 [17]. It comprises two dimensions: professional spiritual sensitivity (Items 1–12) and inner spiritual sensitivity (Items 13–20). A 5‐point Likert scale was used (0 = never, 1 = rarely, 2 = sometimes, 3 = often, and 4 = always). Item scores were summed to obtain a total scale score ranging from 0 to 80, with higher scores indicating greater spiritual sensitivity. The scale can be standardized to a 100‐point grading scale: very low (0–20), low (20.1–40), medium (40.1–60), high (60.1–80), and very high (80.1–100). Standardized score = (original score/maximum score)∗ 100. The Cronbach’s *α* for the overall scale was 0.92, and for the two subscales was 0.92 and 0.88, respectively. EFA indicated that the two dimensions explained 57.62% of the total variance.

### 2.4. Data Collection

We developed an electronic questionnaire on the Questionnaire Star platform, comprising three parts: informed consent, basic information, and the NSSS. The informed consent form was posted on the platform’s homepage, and participants were required to check the consent option before accessing the questionnaire. Instructions were provided at the beginning of each section to facilitate accurate and efficient completion. The questionnaire could be submitted only after all items were completed, and it could be completed once per single IP address.

Data were collected from April 21 to 27, 2025. Before recruitment, the research team explained the purpose, procedures, and significance of the study to the nursing department of Yichang Central People’s Hospital and obtained permission to conduct the survey. With assistance from the nursing department and head nurses, eligible nurses from two hospital branches were invited to participate during scheduled departmental meetings. Before completing the electronic questionnaire independently, participants received a standardized explanation of the study, including its voluntary nature, anonymity, and confidentiality.

For test–retest reliability, 20 nurses were invited by the researchers to complete the questionnaire in person twice, with a 2‐week interval between administrations. A total of 309 and 211 questionnaires were collected from the two hospital branches, respectively. Of these, 303 valid questionnaires (effective response rate 98.06%) were used for EFA, and 205 valid questionnaires (effective response rate 97.16%) were used for CFA.

### 2.5. Statistical Methods

Excel‐formatted data were exported via the Questionnaire Star platform backend and imported into SPSSPRO 1.0.11 for statistical analysis [28]. Means, standard deviations, counts, and percentages were used for descriptive analyses. Critical ratios (CR), item‐total correlations, and homogeneity tests were used for item analysis. An independent samples *t*‐test compared the high group (top 27%) and low group (bottom 27%); items with a CR (t value) < 3.00 or *p* > 0.05 were removed. Item‐total correlations were assessed using Pearson’s correlation coefficients; *r* > 0.40 was considered adequately correlated with the total score. In homogeneity testing, if the removal of an item substantially increased the overall Cronbach’s *α*, the item was deleted [29, 30].

Content validity was evaluated using the content validity index (CVI) via expert review. An item‐level CVI (I‐CVI) ≥ 0.78, a scale‐level CVI (S‐CVI) > 0.90, and an average S‐CVI (S‐CVI/Ave) > 0.90 indicated good content validity [31, 32]. Construct validity was assessed using EFA and CFA. In EFA, a Kaiser–Meyer–Olkin (KMO) value > 0.70 and a Bartlett’s test of sphericity *p* value < 0.05 were considered suitable for factor analysis. Principal component analysis with varimax (maximum variance) orthogonal rotation was used; common factors with eigenvalues > 1 and a cumulative variance contribution > 40% were selected, and items with factor loadings < 0.50 were deleted [33, 34].

In CFA, the model was estimated using the maximum‐likelihood (ML) method, which is appropriate for continuous data. Model fit was assessed using a standard set of indices and widely accepted thresholds: chi‐square/df (*x* ^2^/df) ≤ 5, a root mean square error of approximation (RMSEA) < 0.08, root mean square residual (RMR) < 0.08, comparative fit index (CFI) ≥ 0.90, goodness‐of‐fit index (GFI) ≥ 0.90, and normed fit index (NFI) ≥ 0.90 were considered acceptable [35, 36]. The GFI and NFI are also reported, with the acknowledgment that they are more sensitive to sample size. A model was considered to have acceptable overall fit if the majority of these indices met their respective criteria. If the initial model showed inadequate fit, modification indices were examined, and post hoc adjustments (e.g., allowing correlated errors between items) were made only if they were theoretically justified and did not introduce cross‐loadings. Convergent validity included calculating the average variance extracted (AVE) and composite reliability. If AVE > 0.50 and composite reliability > 0.70, convergent validity was considered acceptable [37]. For discriminant validity, when the √AVE of each factor exceeded the absolute value of its correlations with other factors, discriminant validity was considered good [38].

Cronbach’s *α*, split‐half reliability, and the intraclass correlation coefficient (ICC) from a two‐week test–retest were used to evaluate reliability. Split‐half reliability was evaluated by dividing the 20 NSSS items into two halves (the first 10 and the last 10). Cronbach’s *α* ≥ 0.70 suggested good internal consistency [39]. For test–retest reliability, ICC > 0.80 was interpreted as excellent [40]. Ceiling and floor effects were determined from respondent scores. When the proportions scoring the maximum total (80 points) or the minimum total (0 points) did not exceed 15%, the effects were considered acceptable [41]. The level of statistical significance was set at *α* < 0.05.

### 2.6. Ethics Considerations

This study adhered to the ethical guidelines of the Declaration of Helsinki and was approved by the Ethical Review Board of Yichang Central People’s Hospital (Approval Number: 2024–487‐01). All participants signed an electronic informed consent form in compliance with human subjects research ethics. Study data were kept confidential and used solely for this study. No minors were involved in this study.

## 3. Results

### 3.1. Demographic Information and Characteristics of Respondents

A total of 508 participants were included, of whom 303 were used in EFA and 205 in CFA. There were no statistically significant differences in sex, age, education level, or years of work experience between groups (*p* > 0.05). Baseline characteristics were comparable, supporting their use in EFA and CFA (Table 2).

**TABLE 2:** Distribution of nurses: Demographic information and characteristics (*N* = 508).

Demographic information	EFA (*n* = 303)		CFA (*n* = 205)		*x* ^2^	*p*
	*n*	%	*n*	%		
Sex					1.347	0.246
Female	277	91.42	181	88.29		
Male	26	8.58	24	11.71		
Age (years)					2.852	0.240
< 30	105	34.65	85	41.46		
31–40	147	48.51	85	41.46		
> 40	51	16.83	35	17.07		
Educational level					2.778	0.096
Vocational school	115	37.95	93	45.37		
Bachelor degree	188	62.05	112	54.63		
Years of experience					2.537	0.111
< 10	214	70.63	131	63.90		
≥ 10	89	29.37	74	36.10		

### 3.2. Item Analysis

We used the CR method to assess item discrimination. For each item in the Chinese version of the NSSS, responses were ranked from low to high; the top 27% comprised the high‐score group, and the bottom 27% comprised the low‐score group. An independent samples *t*‐test was used to compare group scores. The results showed that item‐level *p* values were all < 0.01, and CRs ranged from 32.575 to 40.556, exceeding the threshold of 3. These results indicate that the items clearly discriminate between respondents with higher versus lower scores, confirming the scale’s strong discriminatory power across respondent groups. These findings indicated that the Chinese version of the NSSS demonstrated good discrimination across respondent groups. Item‐total correlations were calculated using Pearson’s coefficients. Higher correlations reflect better consistency between each item and the overall scale. The results showed that item‐total correlation coefficients ranged from 0.48 to 0.747, all > 0.40, indicating good consistency. In homogeneity analysis, each item was deleted in turn, and Cronbach’s *α* for the remaining 19 items was computed. Cronbach’s *α* ranged from 0.921 to 0.927 across deletions and did not exceed the overall *α* of 0.927, indicating that no item deletion improved internal consistency; thus, all items contributed positively and were retained. Item analysis supported retaining all items (Table 3).

**TABLE 3:** Item analysis of the Chinese version of the Nurse’s Spiritual Sensitivity Scale (*N* = 508).

Item	*M* (SD)	Item‐total correlation (*p* value)	Critical ratio (*p* value)	Cronbach’s *α* after deletion of terms
Q1	2.41 (1.069)	0.75 (< 0.001)	−32.575 (< 0.001)	0.921
Q2	2.33 (1.04)	0.69 (< 0.001)	−36.04 (< 0.001)	0.922
Q3	2.35 (1.139)	0.68 (< 0.001)	−40.033 (< 0.001)	0.922
Q4	2.39 (1.048)	0.73 (< 0.001)	−34.362 (< 0.001)	0.921
Q5	2.33 (1.094)	0.69 (< 0.001)	−38.708 (< 0.001)	0.922
Q6	2.38 (1.101)	0.66 (< 0.001)	−37.124 (< 0.001)	0.923
Q7	2.31 (1.10)	0.65 (< 0.001)	−36.814 (< 0.001)	0.923
Q8	2.39 (1.074)	0.75 (< 0.001)	−36.301 (< 0.001)	0.921
Q9	2.33 (1.134)	0.69 (< 0.001)	−38.483 (< 0.001)	0.922
Q10	2.31 (1.119)	0.72 (< 0.001)	−39.406 (< 0.001)	0.921
Q11	2.33 (1.05)	0.65 (< 0.001)	−36.076 (< 0.001)	0.923
Q12	2.35 (1.136)	0.66 (< 0.001)	−40.556 (< 0.001)	0.923
Q13	2.47 (1.152)	0.57 (< 0.001)	−36.454 (< 0.001)	0.925
Q14	2.46 (1.129)	0.48 (< 0.001)	−36.32 (< 0.001)	0.927
Q15	2.42 (1.123)	0.63 (< 0.001)	−34.722 (< 0.001)	0.924
Q16	2.4 (1.129)	0.58 (< 0.001)	−34.764 (< 0.001)	0.925
Q17	2.45 (1.12)	0.55 (< 0.001)	−34.742 (< 0.001)	0.925
Q18	2.46 (1.138)	0.63 (< 0.001)	−36.096 (< 0.001)	0.923
Q19	2.47 (1.126)	0.63 (< 0.001)	−36.298 (< 0.001)	0.923
Q20	2.5 (1.152)	0.57 (< 0.001)	−39.593 (< 0.001)	0.925

*Note: M*, mean.

Abbreviation: SD, standard deviation.

### 3.3. Cross‐Cultural Adaptation

During translation and the Delphi process, we partially revised Items 2, 8, 10, and 14, and replaced “religious” with “faith” to better align with Chinese language usage and the clinical work environment. The expert panel agreed that item relevance to the scale was high, and all items were retained.

### 3.4. Content Validity

Content validity was evaluated using I‐CVI, S‐CVI, and S‐CVI/Ave. Six experts in related fields evaluated the relevance of each item to its corresponding dimension using a 4‐point Likert scale (1 = irrelevant, 2 = weakly relevant, 3 = relevant, and 4 = very relevant). The panel comprised one clinical nursing expert, one nursing education expert, one nursing management expert, one nursing expert familiar with the scale structure, one methodology expert, and one translation expert. Among them, 2 held doctoral degrees and 4 held master’s degrees; 4 had senior professional titles and 2 had intermediate professional titles. All had more than 15 years of work experience. In the second round of expert consultation, I‐CVI ranged from 0.833 to 1.000 (≥ 0.78), S‐CVI was 0.90, and S‐CVI/Ave was 0.983 (≥ 0.90), indicating good content validity for the Chinese version of the NSSS. Kendall’s W among the six experts was 0.407 (*p* < 0.001), indicating significant agreement on the scale’s applicability.

### 3.5. Construct Validity

**FIGURE 1:**
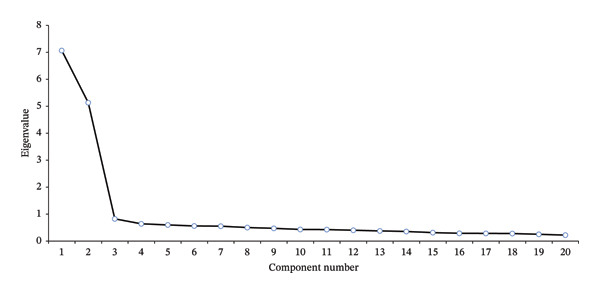
Scree plot of the Chinese version of the Nurse’s Spiritual Sensitivity Scale. The scree plot illustrates the eigenvalues of each factor. Two factors have eigenvalues greater than 1, which account for a significant portion of the total variance.

EFA was conducted on 303 questionnaires. Before EFA, we performed the KMO test and Bartlett’s test of sphericity. The KMO value was 0.958 (exceeding 0.80), and Bartlett’s test statistic was 3798.499 (df = 190, *p* < 0.001), indicating suitability for factor analysis. Based on the principal component analysis (PCA) results and the scree plot (Figure 1), two factors with eigenvalues > 1 (7.067 and 5.133) were extracted. The explained variances were 35.334% and 25.664%, and the cumulative explained variance was 60.999% (exceeding 50%). Item factor loadings ranged from 0.610 to 0.858, all > 0.40. Table 4 shows the factor loadings for each item.

**TABLE 4:** Exploratory factor analysis results of the Chinese version of the Nurse’s Spiritual Sensitivity Scale (*N* = 303).

Dimension/item	Factor loading
*Nurses’ professional spiritual sensitivity*	
Q1—I am able to understand the patient’s/family’s need for spiritual support	0.667
Q2—I am able to understand the patient’s need to perform his/her faith acts	0.848
Q3—I am able to understand the patient’s/family’s need for comfort	0.803
Q4—I am able to understand the frustration of the patient/patient’s family	0.795
Q5—I am able to understand the patient’s remorse and guilt	0.793
Q6—I am able to understand the sadness of the patient	0.61
Q7—I am unable to understand the patient’s/family’s sense of trust in faith during illness	0.737
Q8—I am able to understand the patient’s need for faith education	0.775
Q9—I sympathize with the patient/patient’s family	0.754
Q10—I am able to understand the pain of the patient when he/she is forced to stop his/her faith activities	0.772
Q11—I provide spiritual end‐of‐life care	0.741
Q12—I feel responsible for meeting the patient’s spiritual needs	0.723

*Nurses’ inner spiritual sensitivity*	
Q13—Adherence to spirituality helps me achieve my purpose in life	0.72
Q14—I rarely adhere to doing faith affairs	0.697
Q15—Faith helps me understand the events of life	0.777
Q16—Spirituality is present in all aspects of my life	0.858
Q17—I strive for my spiritual growth	0.818
Q18—I believe that hardships and illnesses secure forgiveness of sins	0.743
Q19—I try to allocate time to meet my spiritual needs	0.831
Q20—Ethical people serve as role models for me	0.762

**FIGURE 2:**
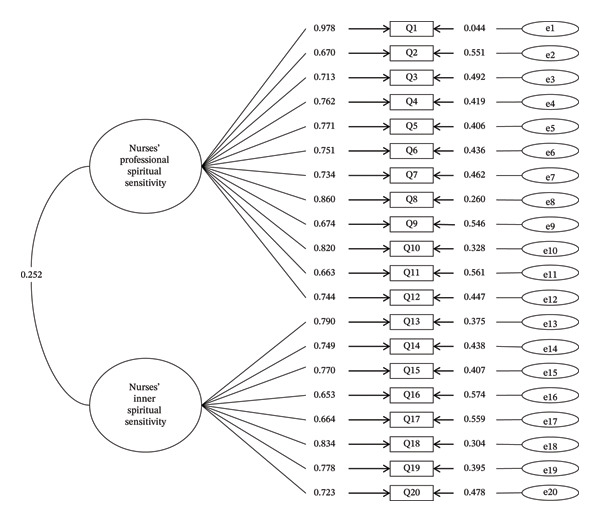
Structural equation model diagram of the Chinese version of the Nurse’s Spiritual Sensitivity Scale.

Next, CFA was conducted on 205 questionnaires. Items 1–12 were assigned to the Nurses’ Professional Spiritual Sensitivity dimension and items 13–20 to the Nurses’ Inner Spiritual Sensitivity dimension; all fit indices met acceptable thresholds (Table 5). As shown in Table 6, standardized factor loadings for items on the two latent dimensions ranged from 0.653 to 0.978; all were ≥ 0.60, indicating strong item loadings on their respective latent factors. The second‐order CFA results are presented in Figure 2. The AVEs for the two dimensions were 0.583 and 0.555 (≥ 0.50), respectively, and the composite reliabilities were 0.943 and 0.909 (≥ 0.70), indicating acceptable convergent validity within the dimensions. The square roots of these values (√AVE = 0.764 for Dimension 1, √AVE = 0.745 for Dimension 2) both exceeded the absolute correlation coefficient between the two dimensions (*r* = 0.223), thereby supporting good discriminant validity.

**TABLE 5:** Model fit indices of the CFA of the Chinese version of the Nurse’s Spiritual Sensitivity Scale (*N* = 205).

Index	Good fit	Acceptable fit	Obtained value	Outcome
*x* ^2^/df	0 ≤ *x* ^2^/df ≤ 3	3 < *x* ^2^/df ≤ 5	1.228	Good
GFI	0.95 ≤ GFI ≤ 1	0.90 ≤ CFI < 0.95	0.927	Acceptable
RMSEA	0 < RMSEA < 0.05	0.05 ≤ RMSEA < 0.08	0.033	Good
RMR	0 ≤ RMR < 0.05	0.05 ≤ RMR < 0.08	0.076	Acceptable
CFI	0.97 ≤ CFI ≤ 1	0.90 ≤ CFI < 0.97	0.986	Good
NFI	0.95 ≤ NFI ≤ 1	0.90 ≤ NFI < 0.95	0.927	Acceptable

*Note:* RMR, root mean square residual.

Abbreviations: CFI, comparative fit index; GFI, goodness‐of‐fit index; NFI, normed fit index; RMSEA, root mean square error of approximation.

**TABLE 6:** Hypothesized confirmatory factor analysis model of the Chinese version of the Nurse’s Spiritual Sensitivity Scale (*N* = 205).

Dimension	Item	Factor loading	Standardization factor loading	*Z* value	SE	*p* value
Nurses’ professional spiritual sensitivity	Q1	1	0.978			
	Q2	0.721	0.67	12.456	0.058	< 0.001
	Q3	0.78	0.713	13.95	0.056	< 0.001
	Q4	0.784	0.762	16.001	0.049	< 0.001
	Q5	0.802	0.771	16.429	0.049	< 0.001
	Q6	0.818	0.751	15.52	0.053	< 0.001
	Q7	0.841	0.734	14.774	0.057	< 0.001
	Q8	0.928	0.86	22.08	0.042	< 0.001
	Q9	0.753	0.674	12.59	0.06	< 0.001
	Q10	0.919	0.82	19.123	0.048	< 0.001
	Q11	0.722	0.663	12.231	0.059	< 0.001
	Q12	0.815	0.744	15.195	0.054	< 0.001

Nurses’ inner spiritual sensitivity	Q13	1	0.79			
	Q14	0.93	0.749	11.485	0.081	< 0.001
	Q15	0.934	0.77	11.878	0.079	< 0.001
	Q16	0.84	0.653	9.719	0.086	< 0.001
	Q17	0.843	0.664	9.914	0.085	< 0.001
	Q18	1.001	0.834	13.149	0.076	< 0.001
	Q19	0.945	0.778	12.026	0.079	< 0.001
	Q20	0.926	0.723	10.98	0.084	< 0.001

Abbreviation: SE, standard error.

### 3.6. Reliability Analysis

Reliability was assessed via internal consistency for the overall scale and both dimensions. The overall Cronbach’s *α* of the Chinese version of the NSSS was 0.927; split‐half reliability was 0.935 for the first half (Items 1–10) and 0.883 for the last half (Items 11–20). Cronbach’s *α* for the nurses’ professional spiritual sensitivity and nurses’ inner spiritual sensitivity dimensions was 0.943 and 0.914, respectively. Overall reliability, split‐half reliability, and Cronbach’s *α* for both dimensions were all ≥ 0.70, indicating excellent reliability of the Chinese version of the NSSS. ICCs for the overall scale and the two dimensions were 0.911, 0.867, and 0.935, respectively; all were ≥ 0.80, indicating excellent test–retest reliability.

### 3.7. Floor/Ceiling Effect

Total scores on the Chinese version of the NSSS were ranked from low to high, and no respondent scored the minimum (0 points) or the maximum (80 points), indicating the absence of floor and ceiling effects.

## 4. Discussion

Spiritual sensitivity is a core competency for nurses in providing effective spiritual care [42]. To evaluate the competence of Chinese nurses in this field, we systematically translated the original NSSS into Chinese and applied it in an empirical assessment of Chinese nurses. Results showed that the Chinese version of the NSSS demonstrated good content and construct validity and high internal consistency, supporting its suitability for assessing spiritual sensitivity in Chinese nursing settings.

During cross‐cultural adaptation, we conducted a comprehensive evaluation of the Chinese version’s validity and reliability to minimize item misinterpretation and distortion [43]. In contrast to some existing tools that are strongly anchored in specific religious contexts or developed for broader populations, our adaptation prioritized conceptual and cultural equivalence in a predominantly secular setting, which is critical for comparable scores across settings and for avoiding construct drift when spirituality is expressed nonreligiously. [44]. Given China’s cultural context, which is dominated by secular beliefs, the expert panel suggested replacing “religious” with “faith” in some items. This revision reflected the diversified understanding of spiritual belief in Chinese society—including institutional religions and noninstitutionalized spiritual pursuits such as Confucian ethics and family values—and ensured consistency with the clause “guaranteeing citizens’ freedom of religious belief” in China’s Regulations on Religious Affairs [45]. Pretesting showed that this cultural adaptation improved applicability: the revised items were understood and accepted by all nurses in the pretest, indicating alignment with the characteristics of Chinese culture and the medical system. This adaptation also improves comparability with instruments that use broader constructs such as “faith” or “belief,” thereby facilitating cross‐study synthesis within the existing literature.

Among all items, Item 13 “Adherence to spirituality helps me achieve my purpose in life,” Item 19 “I try to allocate time to meet my spiritual needs,” and Item 20 “Ethical people serve as role models for me” had high scores, reflecting the correspondence between Chinese cultural values and nursing professional characteristics. Culturally, Item 13 echoes the traditional pursuit of life’s meaning and value; Item 19 aligns with the contemporary emphasis on spiritual nourishment and self‐improvement; and Item 20 directly resonates with the Confucian ideal of moral cultivation. Professionally, these three items collectively reflect translating spiritual pursuits into practical actions: Item 13 supports the construction of professional meaning under heavy workloads, Item 19 indicates self‐regulation in high‐pressure environments, and Item 20 meets the ethical standards emphasized in nursing training and practice. This finding may inform the integration of spiritual beliefs with professional values in nurse training and could guide the creation of practical conditions for the practice of humanistic care as part of efforts to improve nursing quality.

Construct validity of the Chinese version of the NSSS was tested using EFA and CFA. In EFA, we extracted 2 factors, consistent with the original scale [17]. Item factor loadings were ≥ 0.40, indicating sufficient contribution to the scale structure [33]. Item 2, “I am able to understand the patient’s need to perform his/her faith acts,” and Item 16, “Spirituality is present in all aspects of my life,” had the highest loadings: the former reflects professional awareness of patients’ external needs, and the latter reflects self‐awareness of internal spiritual experience—together constituting key manifestations of spiritual sensitivity in Chinese nursing contexts.

CFA results showed that all fit indices met accepted psychometric criteria [46]. Notably, the Turkish version supported a unidimensional structure [47], whereas the Chinese version in the present study retained the original two‐dimensional structure. This difference may stem from cultural contextual factors, including religious composition, nursing education systems, professional values, and exposure to holistic or spiritual care training. Prior studies have highlighted reported cultural traits in Turkish society, such as collectivism, emotional expressiveness, and obedience to authority [48], which may, to some extent, influence the expression or conceptual organization of self‐related spiritual traits. In addition, in Türkiye, where Islam is the predominant religion and religious practices may be more closely integrated into daily life and healthcare experiences, items related to faith and general spiritual sensitivity may be perceived as components of a single underlying construct. By contrast, in China’s relatively secular clinical context, spirituality may be understood more broadly as meaning, dignity, emotional support, hope, family connection, and existential concerns rather than organized religious practices. Therefore, nurses may more clearly differentiate between sensitivity to general spiritual or existential needs and sensitivity to faith‐related practices, which may partly explain why the two‐dimensional structure was retained in the Chinese version. This observation underscores the need to consider cultural frameworks in the cross‐cultural application of psychometric instruments and suggests that measurement invariance testing across cultures represents an important direction for future research. Taken together, these cross‐version differences indicate that, when comparing NSSS scores or findings across instruments or cultural contexts, researchers should account for potential differences in construct operationalization and factor structure rather than assuming measurement equivalence.

The Cronbach’s *α* coefficients of the overall scale and the two dimensions were all above 0.9, indicating excellent internal consistency of the Chinese version of the NSSS [49]. Because participants were recruited from the same hospital, the sample was specific and relatively homogeneous, which may contribute to elevated Cronbach’s *α* values [50]. Sequential item deletion did not increase *α*, supporting retention of all items. In the correlation analysis, item‐total correlations ranged from 0.7 to 0.9, indicating a high degree of fit between each item and the overall scale. Temporal stability was assessed via a two‐week retest, and ICC values for the overall scale and each dimension were above 0.8, indicating excellent test–retest reliability [36]. These findings show that the Chinese version of the NSSS exhibits substantial stability and repeatability in assessing the spiritual sensitivity of Chinese nurses [51].

The Chinese version of the NSSS also has clinical utility in nursing practice. First, it can be used to assess nurses’ self‐perceived spiritual sensitivity and identify areas requiring further support or training. For example, nurses with lower scores on patient‐oriented items may benefit from training in culturally sensitive communication, recognizing patients’ spiritual or existential concerns, and responding appropriately to patients from diverse religious and nonreligious backgrounds. Second, the scale may support reflective practice by helping nurses examine their spiritual awareness, sense of professional meaning, ethical values, and capacity to provide humanistic care. Third, aggregated unit‐level NSSS scores may help nurse managers identify common gaps in spiritual care competence and design targeted educational programs, clinical supervision strategies, or case‐based training activities. The scale may also be used for pre‐ and postprogram assessment to evaluate changes in nurses’ spiritual sensitivity following spiritual care education. However, the NSSS should be used as a supportive and developmental assessment tool rather than as a punitive instrument for individual performance appraisal. These applications suggest that the Chinese version of the NSSS may facilitate integrating spiritual sensitivity assessment into routine nursing education, clinical supervision, and quality improvement activities.

### 4.1. Limitations

Although this study provided preliminary evidence of the reliability and validity of the NSSS in the Chinese cultural context, several limitations should be noted. First, participants were recruited from two hospital branches. No subgroup analyses were conducted by the department. Participants’ ethnicity was not collected, so we could not determine whether the sample included nurses from ethnic minority groups or examine the potential influence of China’s cultural, ethnic, and belief‐related diversity on spiritual sensitivity. Second, several NSSS items explicitly refer to faith‐related activities, such as practices, education, or disruptions to such activities. In Chinese clinical settings, many patients may not engage in organized faith‐based practices. Responses to these items may reflect the infrequency of relevant clinical scenarios rather than nurses’ spiritual sensitivity per se. The current 5‐point Likert scale lacks a “not applicable” option. This limitation may reduce the precision of responses to these items. Third, voluntary participation may have introduced self‐selection bias. Nurses with greater interest or confidence in spiritual care might have been more likely to respond, potentially limiting the sample’s representativeness. Fourth, data collection used a single online platform with IP‐based access restrictions. This method, while efficient, may have excluded nurses with limited digital access or familiarity with the platform, such as older nurses or those working in areas with limited technological infrastructure. Finally, test–retest reliability was assessed in a small subsample. Although the initial estimates were promising, replication in larger samples is warranted. Appropriate criterion measures were unavailable, so criterion‐related validity was not assessed.

Future studies should adopt multicenter, large‐scale stratified sampling designs across different levels of healthcare institutions, departments, regions, and cultural backgrounds to further examine the generalizability of the Chinese version of the NSSS. Cultural adaptation studies involving ethnic minority groups and populations with diverse religious or nonreligious backgrounds should be conducted to improve the scale’s cultural sensitivity and applicability across diverse nursing contexts. Future research should also examine the performance of faith‐related items in predominantly nonreligious clinical settings, including the feasibility of adding a “not applicable” response option, the development of culturally relevant secular spirituality items, and analyses of differential item functioning and measurement invariance. Furthermore, future research should examine the scale’s criterion‐related validity, predictive validity, responsiveness, and utility in guiding interventions to improve spiritual care delivery and patient‐centered outcomes.

## 5. Conclusions

This study showed that the Chinese version of the NSSS demonstrated solid psychometric properties with reliable and valid performance, and it is suitable for evaluating the spiritual sensitivity of Chinese nurses. Nursing managers can use the NSSS to assess nurses’ spiritual sensitivity and then implement targeted interventions based on the assessment results. These actions can systematically enhance nurses’ responsiveness to the spiritual needs of patients and ultimately improve the quality of spiritual care and patient satisfaction.

## 6. Implications for Nursing Management

The validated Chinese NSSS offers potential utility for screening training needs, evaluating educational interventions, and guiding clinical supervision in spiritual care. However, its application requires careful consideration to avoid potential stigma from low scores, misinterpretation of results, and to ensure fair use across nurses with diverse faith or nonfaith backgrounds. Furthermore, the two‐factor structure—distinguishing between professional spiritual sensitivity (directed toward patients) and inner spiritual growth—provides a balanced framework for nursing curricula. Educational programs should aim to develop both dimensions, equipping nurses with the external competencies to respond to patients’ spiritual needs while fostering the internal resilience and self‐care awareness necessary for sustainable, holistic care. Future studies should focus on validating the scale across multisite, more diverse samples, testing its measurement invariance across key subgroups, establishing its convergent validity with related constructs, and evaluating its sensitivity to change following targeted training interventions.

**Nomenclature**
AVE: Average variance extracted
CR: Critical ratio
CVI: Content validity index
CFA: Confirmatory factor analysis
CFI: Comparative fit index
EFA: Exploratory factor analysis
FACES: Face‐to‐face cooperation evaluation scale short version
GFI: Goodness‐of‐fit index
I‐CVI: Item‐level content validity index
ICC: Intraclass correlation coefficient
KMO: Kaiser–Meyer–Olkin
NFI: Normed fit index
NNFI: Nonnormed fit index
NSSS: Nurse’s Spiritual Sensitivity Scale
PCA: Principal component analysis
RMSEA: Root mean square error of approximation
RMR: Root mean square residual
S‐CVI: Scale‐level content validity index
S‐CVI/Ave: Scale‐content validity index/average
SSC: Spiritual sensitivity for children
SSI: Spiritual Sensitivity Inventory (SSI)
SSS: Spiritual Sensitivity Scale
STROBE: Strengthening the Reporting of Observational Studies in Epidemiology

## Author Contributions

Zifeng Li and Xiaojing Zhou were responsible for the study’s conception and design. The data were collected by Zifeng Li, Xiaojing Zhou, Luhuan Yang, Fu Ni, Huiqin Liu, Yongting Wei, Yuanzhi Fu, and Zuyang Xi. Analysis and interpretation of the results were conducted by Zifeng Li, Xiaojing Zhou, and Luhuan Yang. The manuscript was drafted by Zifeng Li, Xiaojing Zhou, and Luhuan Yang, while Zuyang Xi and Fu Ni provided critical revisions.

## Funding

No funding was received for this manuscript.

## Disclosure

All authors read and approved the final manuscript.

## Ethics Statement

This study adhered to the ethical guidelines of the Declaration of Helsinki and was approved by the Ethical Review Board of Yichang Central People’s Hospital (approval number: 2024–487‐01). All participants signed an electronic informed consent form in compliance with human subjects research ethics.

## Consent

Please see the Ethics Statement.

## Conflicts of Interest

The authors declare no conflicts of interest.

## Supporting Information

Additional supporting information can be found online in the Supporting Information section.

## Supporting information


**Supporting Information** STROBE statement.

## Data Availability

The data that support the findings of this study are available from the corresponding author upon reasonable request.
